# Pregnancy and dysfunctional anxiety in women recovered from Covid-19

**DOI:** 10.1192/j.eurpsy.2022.1283

**Published:** 2022-09-01

**Authors:** M. Lagha, G. Hamdi, N. Dhaouadi, S. Chebli, R. Ridha

**Affiliations:** 1 Razi Hospital, Forensic Psychiatry, La Manouba, Tunisia; 2 Abderrahmen Mami Hospital, Preventive Medicine, Ariana, Tunisia

**Keywords:** Anxiety, women, Pregnancy, Covid-19

## Abstract

**Introduction:**

Studies have shown an increasing prevalence of mental health issues in the general population during the COVID-19 pandemic. Among them, pregnant women are a specific population at particular mental risk.

**Objectives:**

The objectives of our study were to assess dysfunctional anxiety in women recovered from COVID-19 and to identify the impact of pregnancy on coronavirus-related dysfunctional anxiety.

**Methods:**

This was a cross-sectional case-control study.

The women in the case group have been infected with Sars-Cov 2, with a benign or pauci-symptomatic clinical form, and cured for one to two months at the time of the study without any post-COVID complications. Women included in the control group have not been infected with Sars-Cov 2 .Anxiety was assessed by the Coronavirus Anxiety Scale (CAS).

**Results:**

In total, we recruited 30 women in the case group and 30 women in the control group.The average age of the case group was 35.8 ±6.8 years versus an average age of 35.3 ± 6.33 years in the control group. In each group, four women were pregnant (13.3%). Nearly one-third of the patients in the case group had a CAS score indicating dysfunctional anxiety probably related to coronavirus (33.3%), with a significant difference with the control group (p=0.026). In the case group, pregnancy was a risk factor for dysfunctional anxiety with p=0.036, OR=19.46 and CI95% = [1.21-314.00].

**Conclusions:**

COVID-19 has a negative impact on perinatal mental health. Specific support for pregnant women is recommanded during the COVID-19 pandemic.

**Disclosure:**

No significant relationships.

